# Supplementary material for: Explainable AI and optimized solar power generation forecasting model based on environmental conditions
Source: PLoS One. 2024 Oct 2;19(10):e0308002. doi: 10.1371/journal.pone.0308002 (PMC11446449; doi:10.1371/journal.pone.0308002)
Supplement: Attachment — Submitted filename: Response to comment.pdf [file pone.0308002.s001.pdf]

Response to Review

Dear Prof. Editor in Chief, PLOS ONE Journal

I would like to thank you very much for the time and effort working on the paper and for the valuable comments and revision which has improved the quality of this paper. I would also like to mention also the English of the paper has been reviewed and edited by Grammarly Package.

In the next, I reply to the comments on the paper. All corrections are highlighted in the original text of the paper in yellow color.

Manuscript Title: “Explainable AI and Optimized Solar Power Generation Forecasting Model based on Environmental Conditions" Article Number: PONE-D-24-10041

Reviewer Comment	Response	Page Number
**Reviewer # 1**		
1)In the introduction, please correct the statement that which increases the power generation, and which decreases the power generation, “Temperature and solar irradiance changes can cause power generation to increase or decrease suddenly.”	Thank you for your comment, it is revised and updated	Page 2-5
2)Please give further details of “original model”, which is mentioned in the abstract.	Thank you. The clarification was added to the abstract<br>" These results improve the performance of the original model that acts without hyper-parameter optimization "	Page1
3)Give a glimpse of software/methodology of the proposed model in the abstract.	The clarification was added to the abstract.<br>The proposed model is implemented utilizing Tensorflow and Keras within the Google Colab environment.	Page1
4)Keywords are Vogue. Ideally, the keywords should be between 4-6. I suggest the following suitable keywords for this work. “1)	Thank you for your recommendation, it is revised and updated	Page 2

Forecasting of solar power generation, ,2) Equilibrium Optimizer, 3) Long short-term memory (LSTM), 4) explainable artificial intelligence (XAI), 5) Local Interpretable and Model-independent Explanation (LIME), 6) Deployment of solar power plants.		
5) Please provide the source data and code of the study.	The source data is cited on page 10 via the hyperlink in reference number 36.	Page 10
6)A continuity is missing for starting of third paragraph of introduction. So, please merge it with the 2nd paragraph.	Thank you for your comment, it is revised and the introduction has been updated	Page 2-5
7)Introduction section could be improved by maintain a good flow of area of study, major literature and contributions.	Thank you for your comment, it is revised and the introduction has been updated	Page 2-5
8)How does the various MPPT techniques impacts the proposed study? Can the MPPT be investigated in this work?	The present study doesn't consider the MPPT techniques, it may considered in the future work	Page 32
9)Conclusion is needed to be improved by ignoring the repeated problem statement.	Thank you for your comment, it is revised and the conclusion has been updated	Page 31
10)In the present study, only the input parameters for generation of power is considered. How could the study go if output loads considered? And further, is	The proposed Work without electrical loads, and in the case of electrical loads, we will take the load distribution and apply the proposed model to it.	Page 32

<table>
<tr><td>this work applied to constant power source generating systems such as small hydro-power systems, if yes, then pls give a future direction by considering the following latest references on constant speed-driven generating stations.
https://doi.org/10.1016/j.renene.2022.06.051
https://doi.org/10.1016/j.rineng.2024.101761</td><td></td><td></td></tr>
<tr><td colspan="3">Reviewer # 2</td></tr>
<tr><td>he submitted manuscript titled 'Explainable AI and Optimized Solar Power Generation Forecasting Model based on Environmental Conditions' presents a study of using optimized long short term memory algorithm to predict energy production from the sun using solar irradiance based on data from India available on Kaggle. However, the link provided at [36] did not lead to dataset in question.</td><td>It works , this is the screen shot of the link site
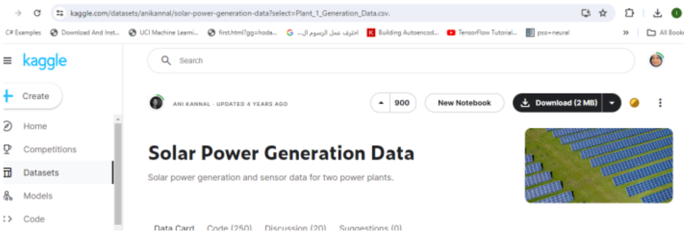
</td><td></td></tr>
</table>

The authors are advised to review all references, and update review of literature with recent publications from 2022-2024 regarding short-and long-term solar irradiance forecasting. While also subjecting the data to greater scrutiny.	Thank you for your comment, it is revised and the references has been updated	Pages 32-35
The novelty of the work as described in the manuscript is insufficient to merit further consideration. The authors must improve the state of the art of solar energy production forecasting using LSTM by a significant margin, and better highlight their contributions in the manuscript.	thank you for your comment, but our novelty and the contributions in the paper are:<br>• Deep learning models might not be as accurate because they use traditional optimization methods to find the best internal parameters. These techniques can get stuck in local minima, which leads to finding parameters that aren't as good as they could be. So, this paper solves these problems by adding the LSTM and the equilibrium optimization (EO) to the suggested model. This model is then used to show correctly how solar output power is related to external factors.<br>• Applying the Equilibrium Optimizer (EO) algorithm for tuning the hyper-parameters the LSTM to enhance the performance of the forecasting, the performance was evaluated in this paper after the application of EO with LSTM.<br>• Applying PSO optimizer for comparing its results with EO optimizer.<br>• The utilization of LSTM for effective exploration of the search space without being trapped in local optima areas.<br>• To understand the forecasting results. XAI's approach called LIME has been applied to explain the performance of the proposed deep learning model, the XAI explained the most important environmental condition that affects the model's forecasting results.<br>• The propped X-LSTM-EO model proposes a common, accurate model that predicts well under many environmental scenarios. It mitigates PV power generation unpredictability and safely integrates large-scale PV power generation into micro grids, lowering operational costs and boosting efficiency and safety.	

The figures also need considerable effort to improve quality and readability, please avoid using screen shots or screen grabs and rely on professional graphics.	Thank you for your comment, it is revised and updated	
The using of english language should also be reviewed. There are some instances where the meaning is not properly conveyed to the reader. While it doesn't pose a challenge to native speakers, it would be beneficial for the manuscript to undergo a thorough review of academic english language usage.	Thank you for your comment, it is revised and updated	
Reviewer # 3		
The study developed a LSTM based model to forecast the solar power using explainable AI. The study is interesting and can be consider after strictly revision of following points.<br><br>1) Reduce the length of Abstract	Thank you for your comment, it is revised and the abstract has been updated	Page 1
2) As the LSTM used in the work, the literature of LSTM must be focused at maximum. study based on contrast models like GRU and BILSTM should also discussed in the literature.	Thank you for your comment, it is revised and the introduction has been updated	Page 2-5

For reference, following studies should consider: https://www.tandfonline.com/doi/abs/10.1080/15435075.2022.2143272 https://link.springer.com/article/10.1007/s12145-023-01020-9 https://ajse.aiub.edu/index.php/ajse/article/view/212		
3) Describe novelty and reason of development of such model only.	Thank you for your comment, but our novelty and the contributions in the paper are:<br>• Deep learning models might not be as accurate because they use traditional optimization methods to find the best internal parameters. These techniques can get stuck in local minima, which leads to finding parameters that aren't as good as they could be. So, this paper solves these problems by adding the LSTM and the equilibrium optimization (EO) to the suggested model. This model is then used to show correctly how solar output power is related to external factors.<br>• Applying the Equilibrium Optimizer (EO) algorithm for tuning the hyper-parameters the LSTM to enhance the performance of the forecasting, the performance was evaluated in this paper after the application of EO with LSTM.<br>• Applying PSO optimizer for comparing its results with EO optimizer.<br>• The utilization of LSTM for effective exploration of the search space without being trapped in local optima areas.<br>• To understand the forecasting results. XAI's approach called LIME has been applied to explain the performance of the proposed deep learning model, the XAI explained the most important environmental condition that affects the model's forecasting results.<br>• The propped X-LSTM-EO model proposes a common, accurate model that predicts well under many environmental scenarios. It mitigates PV power generation unpredictability and safely integrates large-scale PV power generation into micro grids, lowering operational costs and boosting efficiency and safety.	

4) Section-1 have the literature & contributions based on studies. Why separate section -2 (related work) ?	Thank you for your comment, it is revised updated	Page 2- 5
5) Point wise conclusion can be provided.	Thank you for your comment, it is revised and the conclusion has been updated	
6) Limitation of Work OR challenges??	Thank you for your valuable comment,<br>The proposed model in this paper exhibits high prediction accuracy under various environmental conditions, demonstrating its universality and accuracy. It reduces uncertainty in PV power generation and safely integrates large-scale PV power generation into micro grids, reducing operating costs and improving efficiency and safety. It also introduces a new PV power generation forecast research direction: clustering data and noise reduction can reduce uncertainty.<br>The research in this paper does not account for harsh weather conditions such as thunderstorms, sand, and dust.<br>This part added to the conclusion section.	Page 31
7) Provide papers of last 5 years in literature. some are from year 2015,2017. Refer comment 2	Thank you for your comment, it is revised updated	
8) Discussion must consider the topologies and complexities of the models.	Thank you for your comment. First, Table 9 displays the calculation of the training time for the three examined models throughout the training process. Which indicates the complexity of each one.<br><br>Second, the section of the experiment, especially Table 7, page 21, explains the topology of our proposed model-based LSTM. Furthermore, the other techniques—linear regression and decision trees—do not resemble LSTM in their topology, hence it isn't very sensible to compare the topologies.	Table 9 page 23
9) Same for Comparison with different models.	Thank you for your comment, the Comparison exists in section 5.1 , table 13	Page 28
