# Supplementary material for: Explainable AI and optimized solar power generation forecasting model based on environmental conditions
Source: PLoS One. 2024 Oct 2;19(10):e0308002. doi: 10.1371/journal.pone.0308002 (PMC11446449; doi:10.1371/journal.pone.0308002)
Supplement: Attachment — Submitted filename: Response to comments 3-5.docx [file pone.0308002.s002.docx]

**Response to Review**

**Dear Prof. Editor in Chief, PLOS ONE Journal**

I would want to express my gratitude to you for the time and effort that you put into working on the paper, as well as for the insightful comments and revisions that have contributed to the overall improvement of the quality of this paper.

The following is a response to the comments that were made on the paper. The original text of the document has been highlighted in yellow to indicate that all corrections have been found.

**Manuscript Title:** “Explainable AI and Optimized Solar Power Generation Forecasting Model based on Environmental Conditions" Article Number: PONE-D-24-10041R1

| **Reviewer Comment** | **Response** | **Page Number** |
| --- | --- | --- |
| **Reviewer # 3** | | |
| 1) Since the authors mention the MPPT is a future work, then they should give at least few reference for it. I suggest authors refer the dedicated review journals and cite appropriate publications. <https://www.sciencedirect.com/journal/renewable-and-sustainable-energy-reviews> | Thank you for your comment, it is revised. the text is updated and reference 49 is added | Page 30.  Line 3:6 |
| 2) My earlier comment (comment 10) is partially addressed in the conclusion without proper citations in the conclusion for "distribution of electrical loads". | Thank you for your comment, it is revised. the text is updated and reference 50 is added | Page 30  Lines 3:6 |
| 3) Should give the page numbers for the revised manuscript and line number as you mention in the Comments & Response sheet. | Take into consideration |  |
| 4) Please check the numbering for section and subsections properly and also provide the numbering for every subheadings. Wrong numbering is given for sections 4 and 5. | Thank you for your comment, it is revised and updated |  |
| 5) Provide a table for acronyms. | List of Acronyms Table is added | Page 2 |
