# Supplementary material for: Explainable AI and optimized solar power generation forecasting model based on environmental conditions
Source: PLoS One. 2024 Oct 2;19(10):e0308002. doi: 10.1371/journal.pone.0308002 (PMC11446449; doi:10.1371/journal.pone.0308002)
Supplement: Attachment — Submitted filename: Response to Review 11-7-2024.docx [file pone.0308002.s003.docx]

**Response to Review**

**Dear Prof. Editor in Chief, PLOS ONE Journal**

I would like to thank you very much for the time and effort working on the paper and for the valuable comments and revision which has improved the quality of this paper.

In the next, I reply to the comments on the paper. All corrections are highlighted in the original text of the paper in yellow color.

**Manuscript Title:** “Explainable AI and Optimized Solar Power Generation Forecasting Model based on Environmental Conditions" Article Number: PONE-???? -> الرقم مش واضح

| **Reviewer Comment** | **Response** | **Page Number** |
| --- | --- | --- |
| **Reviewer # 4** | | |
| 1-The methodology section, particularly the description of the Equilibrium Optimizer (EO) algorithm and its implementation lacks clarity and detailed steps. The current narrative could benefit from a more structured presentation to facilitate comprehension. Including pseudocode or a flowchart would help in understanding the sequential steps of the algorithm. By visualizing the process, readers can better grasp the logic and flow of the EO algorithm | Thank you for your valuable comment , the algorithm has been revised and updated | Page 11, 12 |
| More literature review is need for comparison. Add more studies and make comparative analysis with recent works, here are some examples:  a.Bukhari SM, Moosavi SK, Zafar MH, Mansoor M, Mohyuddin H, Ullah SS, Alroobaea R, Sanfilippo F. Federated transfer learning with orchard-optimized Conv-SGRU: A novel approach to secure and accurate photovoltaic power forecasting. Renewable Energy Focus. 2024 Mar 1;48:100520.  b.Abou Houran M, Bukhari SM, Zafar MH, Mansoor M, Chen W. COA-CNN-LSTM: Coati optimization algorithm-based hybrid deep learning model for PV/wind power forecasting in smart grid applications. Applied Energy. 2023 Nov 1;349:121638.  c.Khan UA, Khan NM, Zafar MH. Resource efficient PV power forecasting: Transductive transfer learning based hybrid deep learning model for smart grid in Industry 5.0. Energy Conversion and M-- | Thank you for your comment. thee updates are doe as follows:   - the literature review part is revised & updated - the table 1 is added, it contains "Related works for solar power prediction based on AI tools" - Reference 34,35 ad 36 are added to the paper | Pages:  5, 6  7  36 |
| 3-The data preparation phase mentions combining two datasets, but it lacks specifics on how missing values, outliers, and discrepancies between the datasets were handled. Effective data preprocessing is crucial for the reliability of the model, and a detailed account of these steps is essential. Providing detailed steps on data cleaning, handling missing values, and addressing any discrepancies between the two datasets before integration would ensure transparency and reproducibility of the results. | Thanks for your comment.  Before data preparation, we do analysis for the data in section 3 which named " Dataset description and analysis", this step proved the normality of the data and it is clear in table 4 which preset the count , mean, std , min, ad max values of the dataset | Page 12 , 13 |
| 4-The explanation of evaluation metrics like R2, RMSE, MAE, COV, and EC lacks context and examples of how these metrics are calculated and interpreted. These metrics are pivotal in assessing the model’s performance, and a clear understanding of their computation and significance is necessary. Including a brief explanation with examples for each evaluation metric to illustrate how they are calculated and what they signify in the context of the model’s performance would enhance the manuscript's clarity and educational value. | Thank you for comment. Section 4.3 called "Model evaluation" explains in details the evaluation metrics | Page 22, 23 |
| 5-While the paper compares the proposed model with Decision Tree and Linear Regression, it would benefit from a comprehensive comparative analysis with other advanced models, such as Gradient Boosting or other deep learning architectures.  This expanded comparative analysis would provide a broader perspective on the proposed model’s performance and demonstrate its robustness and competitiveness against a wider array of contemporary methods. | Thank you for your valuable comment which proof the robustness and competitive of our proposed model.   - We apply Gradient Boosting algorithm, the results added to table 9. the results of Gradient Boosting ensure the robustness of our work - The optimizer algorithm (EO)is used to get the optimal architecture of the deep learning (LSTM) | Page 25 |
| 6-Ensuring consistent use of terminology throughout the manuscript is crucial for clarity. For example, the term "Equilibrium Optimizer" should be uniformly referred to as EO throughout the text. | Thank you for your valuable comment, it is revised & updated | All the paper |
